# Tauopathy strains differentially replicate *in vitro* in the presence of mutant tau monomer

**DOI:** 10.1016/j.nbd.2025.107052

**Published:** 2025-08-06

**Authors:** Christine K. Brown, Matthew P. Frost, Sara A.M. Holec, William W. Seeley, Lea T. Grinberg, Steven H. Olson, Amanda L. Woerman

**Affiliations:** a Department of Biology and Institute for Applied Life Sciences, University of Massachusetts Amherst, Amherst, MA, USA; b Department of Microbiology, Immunology, and Pathology, Prion Research Center, Colorado State University, Fort Collins, CO, USA; c Departments of Neurology and Pathology, University of California, San Francisco, San Francisco, CA, USA; d Conrad Prebys Center for Chemical Genomics, Sanford Burnham Prebys Medical Discovery Institute, San Diego, CA, USA

**Keywords:** Argyrophilic grain disease, Corticobasal degeneration, Frontotemporal lobar degenerative diseases, Glial globular tauopathy, Progressive supranuclear palsy, Tau strains

## Abstract

Tauopathies are a group of neurodegenerative diseases caused by misfolding of the protein tau into a β-sheet rich conformation. By inducing misfolding of additional tau monomers, these pathogenic fibrils replicate and spread progressive disease throughout the brain. While frontotemporal lobar degenerative diseases (FTLDs) – including argyrophilic grain disease, corticobasal degeneration, globular glial tauopathy, and progressive supranuclear palsy – are caused by this same underlying molecular process, each disease is defined by a unique clinical and neuropathological presentation. This phenomenon is explained by the strain hypothesis, which proposes that the conformation tau misfolds into determines which disease a patient will develop. Indeed, robust structural and biological data indicate that tau misfolds into conformational arrangements specific to each disease. While these findings are highly impactful for understanding similarities and differences between tauopathies, they have yet to be harnessed to develop a definitive ante-mortem diagnostic. Working toward the goal of disease-specific diagnostics, we created a panel of tau bioreporter cell lines expressing a fragment of human tau fused to yellow fluorescent protein (YFP). Using point mutations designed to interfere with tau misfolding into specific conformations, we quantified YFP-positive puncta after incubating cells with tau fibrils from FTLD patient samples to establish a strain-specific profile for each tauopathy. Not only can we use this approach to differentiate between human tau strains, but we also show that the tau strain found in a commonly used mouse model exhibits properties that significantly differ from those seen in human patients.

## 1. Introduction

In a group of neurodegenerative disorders, referred to as tauopathies, the soluble and intrinsically disordered protein tau misfolds into a β-sheet-rich structure capable of inducing additional protein misfolding. As misfolded tau accumulates into larger fibrils in neurons and astrocytes, these inclusions spread throughout the brain leading to progressive degeneration and worsening of clinical signs. While this unifying disease mechanism is the underlying cause of several frontotemporal lobar degenerative diseases (FTLDs), including argyrophilic grain disease (AGD), corticobasal degeneration (CBD), globular glial tauopathy (GGT), and progressive supranuclear palsy (PSP), each disease is defined by a unique clinical presentation, as well as type and distribution of tau inclusions in the brain. For example, AGD patients typically develop a very late onset of cognitive impairment, dementia, and some behavioral abnormalities, which are caused by tau misfolding into argyrophilic grains (Grinberg and Heinsen, 2009). By comparison, PSP syndrome patients classically present with the vertical supranuclear gaze palsy, as the name suggests, along with gait freezing and a speech or language disorder (Höglinger et al., 2017). Neuropathological lesions in PSP patients include neurofibrillary tangles with a globose appearance and tufted astrocytes (Dickson et al., 2007).

Attempts to explain how the tau protein can cause a large group of FTLDs with their own set of clinical and neuropathological criteria drew on research from the prion field. This same phenomenon was first observed for the prion protein (PrP), which misfolds from the cellular conformation (PrP^C^) into the pathogenic conformation (PrP^Sc^) in fatal neurodegenerative diseases including Creutzfeldt-Jakob disease (CJD), Gerstmann-Straussler-Scheinker disease, and fatal familial insomnia. Explaining this phenomenon, Richard Bessen and Richard Marsh proposed the strain hypothesis in 1992, which argues that PrP^Sc^ misfolds into distinct shapes, or conformations, and that the biochemical properties associated with each unique structure dictate the biological consequences in an affected individual (Bessen and Marsh, 1992a; Bessen and Marsh, 1992b).

To investigate the presence of tau strains using an *in vitro* system, Marc Diamond’s laboratory established a bioreporter cell line using human embryonic kidney (HEK293) cells to express a fragment of the tau protein fused to yellow fluorescent protein (YFP) *via* a flexible linker (Sanders et al., 2014). This cell line, Tau4RD(LM)-YFP, was highly valuable in demonstrating that different sources of tau fibrils, both synthetically derived from recombinant protein or isolated from human patient samples, were capable of inducing tau inclusions with distinct morphologies. For example, cells incubated with tau isolated from CBD patient samples largely induced disordered inclusions whereas the PSP patient samples predominantly induced mosaic inclusions.

While these experiments were largely informative about tau strain biology, a major drawback was the use of a tau fragment that failed to capture the complexity of tau biology. In humans, the tau gene, *MAPT*, is alternatively spliced to produce 6 different isoforms (Goedert et al., 1989; Goedert et al., 1992), including variable expression of either 3 or 4 repeats in the repeat domain (RD) near the C-terminal end of the protein (Andreadis et al., 1992). Due to selective isoform recruitment into neuropathological lesions in FTLD patients, tauopathies are now sub-categorized into the 3R tauopathy Pick’s disease (PiD) (Delacourte et al., 1996), the 4R tauopathies AGD, CBD, GGT, and PSP (Flament et al., 1991; Ksiezak-Reding et al., 1994), and the mixed 3R and 4R tauopathies Alzheimer’s disease (AD) and chronic traumatic encephalopathy (CTE) (Goedert et al., 1992; Hong et al., 1998; Schmidt et al., 2001). Investigating the role of this isoform specificity in disease progression, we built an expanded panel of the HEK293T bioreporter cell lines to show that that replication or amplification of tau fibrils isolated from FTLD patient samples is isoform-dependent (Woerman et al., 2016). These biological data were subsequently confirmed by cryo-electron microscopy (cryo-EM) studies, which provided definitive support for the presence of conformationally distinct tau strains in human tauopathy patient samples (Falcon et al., 2018; Falcon et al., 2019; Fitzpatrick et al., 2017; Shi et al., 2021; Zhang et al., 2020).

Drug discovery efforts targeting prion diseases have shown that effective small molecules can exhibit strain specificity (Berry et al., 2013; Giles et al., 2017). These findings highlight the need to use ante-mortem diagnostics to identify not only misfolded protein but also protein conformation in neurodegenerative disease patients to improve therapeutic outcomes in clinical trials. Given that there are currently no definitive diagnostics available to support ante-mortem diagnosis of FTLD, we sought to use the emerging tau cryo-EM structures as a starting point for developing disease-specific bioreporter cell lines capable of differentiating between the 4R tauopathies. In analyzing the 4R tau cryo-EM structures, we identified a handful of mutations that we hypothesized would select for replication of tau fibrils into one or two known conformations while blocking amplification of others. Using this novel panel of cell lines, we generated an infectivity profile for tau fibrils isolated from AGD, CBD, GGT, and PSP patient samples, which can be more broadly used for strain typing. These data can also serve as a critical starting point in developing strain-specific diagnostic tools for FTLD patients.

## 2. Results

### 2.1. The N279K mutation selects for replication of CBD tau over other 4R tauopathies

As discussed above, we previously used HEK293T reporter cell lines expressing the RD of tau fused to YFP *via* a flexible linker to investigate the role of individual isoforms in tau strain replication *in vitro* (Woerman et al., 2016). Building on those findings, we sought to establish a panel of 4RD cell lines capable of further discerning between the 4R tauopathies based on their ability to replicate using mutant substrates. While our previous cell lines were limited to expression of the RD of 3R tau, 4R tau, or both, here we extended the fragment of 4RD tau expressed in our cell lines to encompass residues 244–380 (based on the longest tau isoform) to include the full templating region reported in the 4R tau fibril structures isolated from human patient samples (Shi et al., 2021; Zhang et al., 2020).

**Fig. 1.**
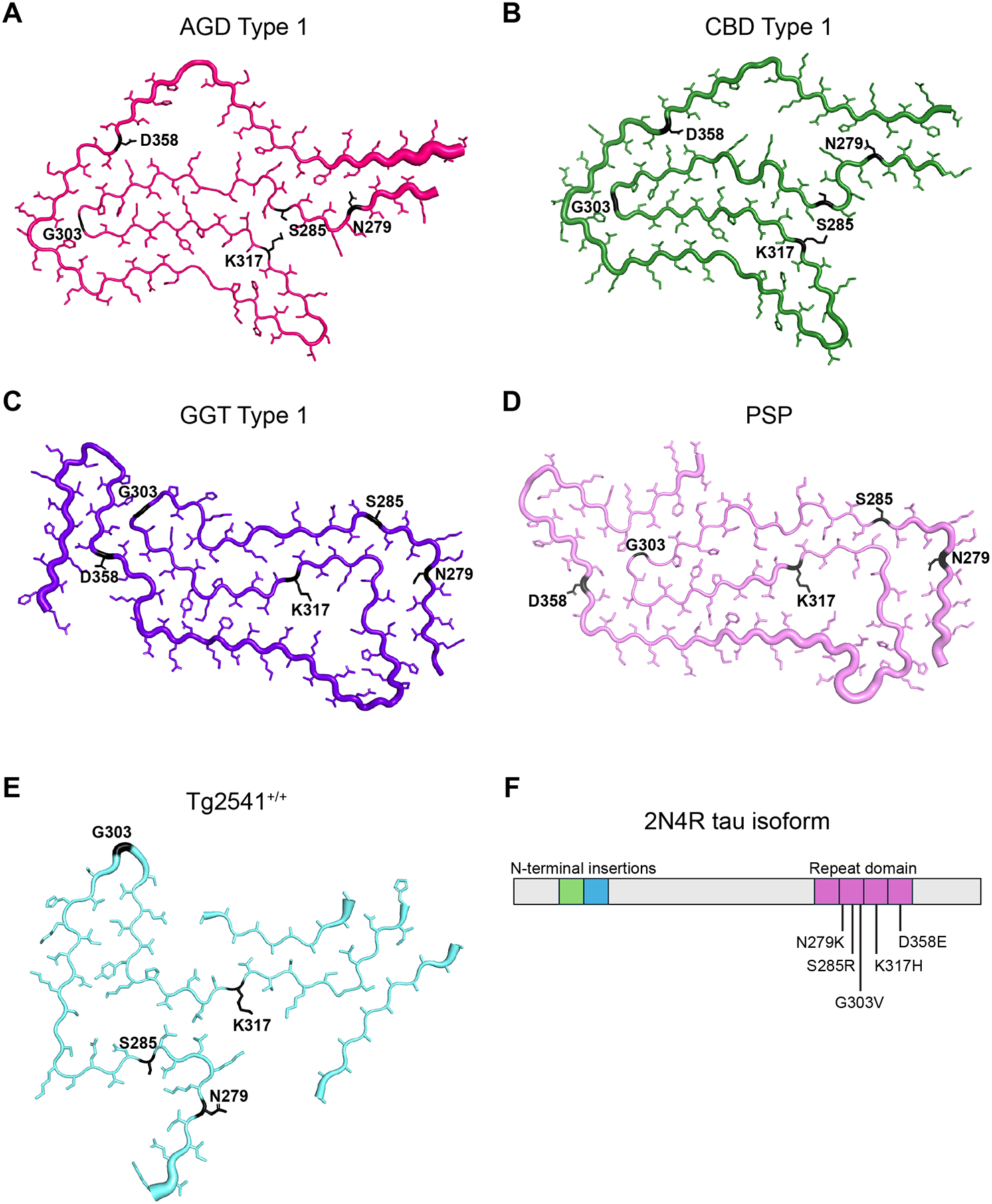
Mutagenesis of 4-repeat tau across five tauopathy strains. Four-repeat tau misfolds into distinct fibril conformations in patients with (A) argyrophilic grain disease (AGD; pink), (B) corticobasal degeneration (CBD; green), (C) glial globular tauopathy (GGT; purple), and (D) progressive supranuclear palsy (PSP; lavender). These structures differ from (E) the tau fibrils that spontaneously develop in the brains of Tg2541^+/+^ mice, which express 0N4R tau with the P301S mutation (light blue). Tau 4RD residues targeted with mutations tested here (N279K, S285R, G303V, K317H, and D358E) are shown in black. Notably, residue D358 is outside the templating region of the Tg2541^+/+^ fibril structure. Tau fibril structures previously reported as AGD type 1 (PDB ID: 7P6D) (Shi et al., 2021), CBD type 1 (PDB ID: 6TJO) (Zhang et al., 2020), GGT type 1 (PDB ID: 7P66) (Shi et al., 2021), PSP (PDB ID: 7P65) (Shi et al., 2021), and Tg2541^+/+^ (PDB ID: 8Q96) (Schweighauser et al., 2023). (F) Diagram of the longest isoform of tau (2N4R) and the location of the mutations tested within the repeat domain of the protein.

The selection of mutations tested here was done using two approaches. In the first, *MAPT* mutations associated with a 4R tauopathy were selected from the literature based on their potential to interfere with known tau fibril conformations. These include the N279K (Delisle et al., 1999), S285R (Fujioka et al., 2015), and G303V mutations (Ros et al., 2005). In the second approach, novel mutations were designed based on their potential to interfere with tau misfolding into at least one of the known fibril structures, as determined by the introduction of steric clashing visualized using point mutation mutagenesis modeling in PyMol software. These include the K317H and D358E mutations. Fig. 1 shows the location of the five residues selected for mutagenesis (highlighted in black) on the reported cryo-EM structures of tau fibrils isolated from deceased human patient samples neuropathologically diagnosed with (A) AGD, (B) CBD, (C) GGT, and (D) PSP. For comparison, the fibril structure of tau isolated from Tg2541^+/+^ mice, which express 0N4R human tau with the P301S mutation (Allen et al., 2002), is shown in Fig. 1E. All five cryo-EM structures were reported by Michel Goedert and Sjors Scheres (Schweighauser et al., 2023; Shi et al., 2021; Zhang et al., 2020).

**Fig. 2.**
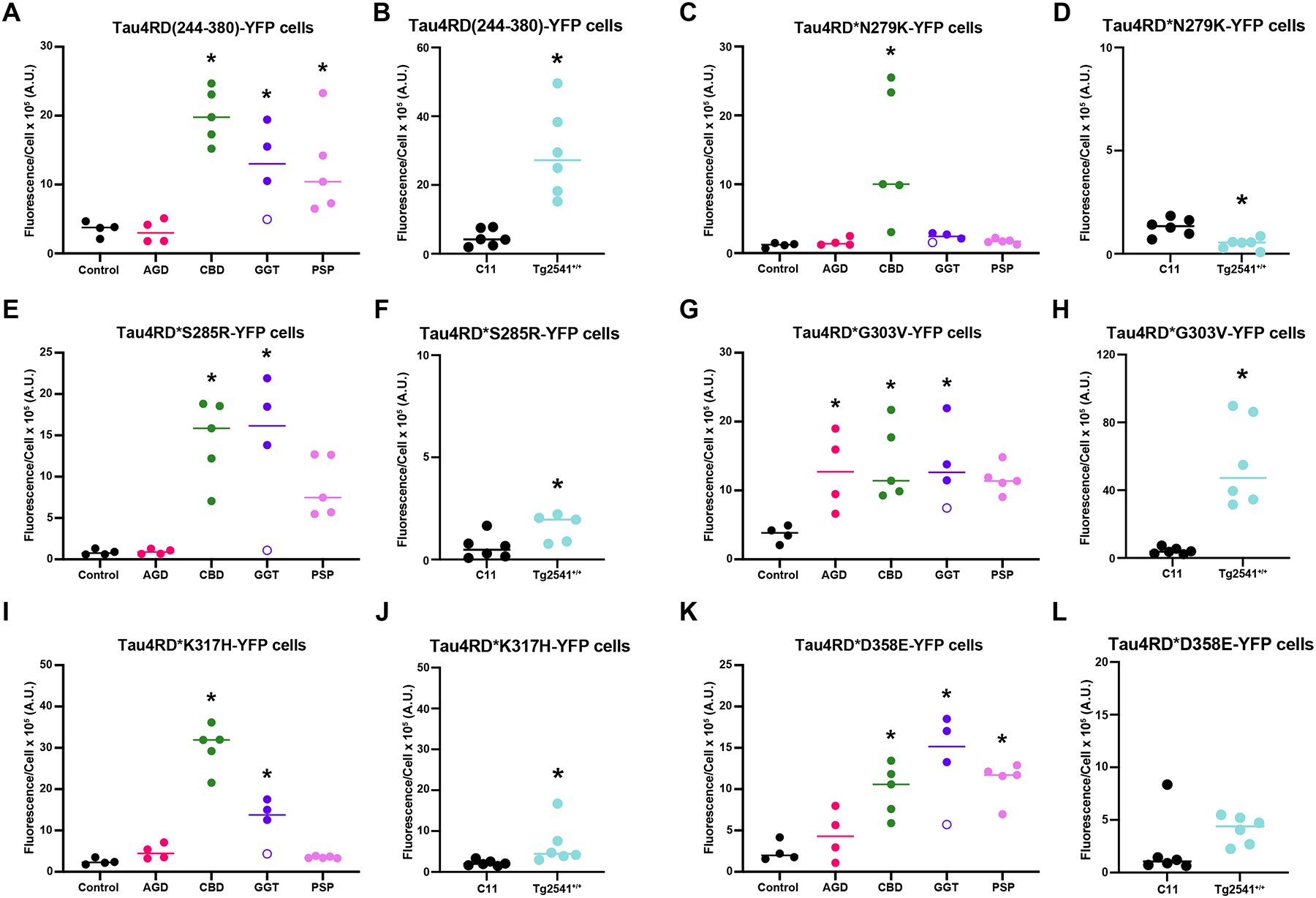
*MAPT* mutations selectively inhibit 4R tau prion strain replication. Tau prions were isolated from control, DLB, AGD, CBD, GGT, and PSP human patient samples, as well as a pooled homogenate from aged Tg2541^+/+^ mice *via* sodium PTA precipitation. Pellets from each sample were incubated for 4 d with cells expressing the Tau4RD(244–380)-YFP construct with either (A & B) the WT sequence or the (C & D) N279K, (E & F) S285R, (G & H) G303V, (I & J) K317H, or (K & L) D358 mutations. (A, C, E, G, I, & K) Quantification of cell infection using control and DLB (*n* = 2 for each, black), AGD (*n* = 4, pink), CBD (*n* = 5, green), GGT (*n* = 4, purple), and PSP (*n* = 5, lavender) samples shows total fluorescence measurement across all aggregates normalized to cell count (× 10^5^ artificial unites [A.U.]). Data from patient sample GGT4 is shown as an open circle. (B, D, F, H, J, & L) Quantification of cell infection using control patient sample C11 (*n* = 6 technical replicates, black) and Tg2541^+/+^ (*n* = 6 technical replicates, light blue). * = *P* < 0.05. All values are shown in Supplemental Table 3.

The initial Tau4RD-YFP cell lines expressed both the P301L and V337M mutations (Woerman et al., 2015; Woerman et al., 2016). To remove the confounding effects of these two mutations on our data, we first generated Tau4RD(244–380)-YFP cells that express WT 4RD tau (Figs. 2A & B; Supplemental Table 3; representative images shown in Fig. S1). Using brain homogenates from two control patient samples (individuals who died for reasons other than a neurodegenerative disease) as well as from two dementia with Lewy body (DLB) patient samples, we isolated aggregated protein using our established sodium phosphotungstic acid (NaPTA) protocol, and found that none of the four control samples were able to infect the Tau4RD(244–380)-YFP cells (cell-line specific assay conditions reported in Supplemental Table 2). By comparison, the five CBD (*P* = 0.0003), four GGT (*P* = 0.0479), and five PSP patient samples (*P* = 0.0423) were all able to replicate using the WT protein (Fig. 2A). The four AGD samples did not significantly infect the WT-expressing cells (*P* > 0.9999), though this may be due to the small grain-shaped aggregates induced by the AGD samples, consistent with our previous findings (Woerman et al., 2016). Data were analyzed using a one-way ANOVA [*F* = 9.962, *P* = 0.0002, degrees of freedom in the numerator (DFn) = 4, degrees of freedom in the denominator (DFd) = 17]. By comparison, tau fibrils isolated from the Tg2541^+/+^ mouse brain homogenate induced robust tau misfolding in the cells compared to patient sample C11 (Fig. 2B; *P* = 0.0010). Normality tests for both datasets are shown in Fig. S2.

We then tested the ability of the same set of patient samples to replicate in cells expressing 4RD(244–380) tau containing the N279K mutation (Fig. 2C; Supplemental Table 3; representative images shown in Fig. S1). This mutation, which has been shown to alter tau isoform splicing leading to an increase in 4R tau expression (Dawson et al., 2007; Hasegawa et al., 1999; Wren et al., 2015), as well as cause tau localization to the nuclear compartment (Ritter et al., 2018), has been identified in patients with familial FTLD (Arima et al., 2000; Delisle et al., 1999; Tsuboi et al., 2002; Yasuda et al., 1999). Again, the negative control samples were unable to induce tau misfolding in the reporter cells. However, when we tested the human tauopathy samples, only the CBD patient samples were able to replicate using N279K tau (*P* = 0.0021). The AGD (*P* = 0.9997), GGT (*P* = 0.9884), and PSP (*P* = 0.9991) samples failed to generate YFP-positive tau puncta in the cells. Data were analyzed using a one-way ANOVA (*F* = 7.051, *P* = 0.0015, DFn = 4, and DFd = 17). Moreover, when control patient sample C11 was compared with seeding activity measured from the Tg2541^+/+^ brain homogenate pool, the Tg2541^+/+^ samples showed a significant decrease in infectivity (*P* = 0.0023), though this difference was not biologically different from control (Fig. 2D). Normality tests for both datasets are shown in Fig. S2.

**Fig. 3.**
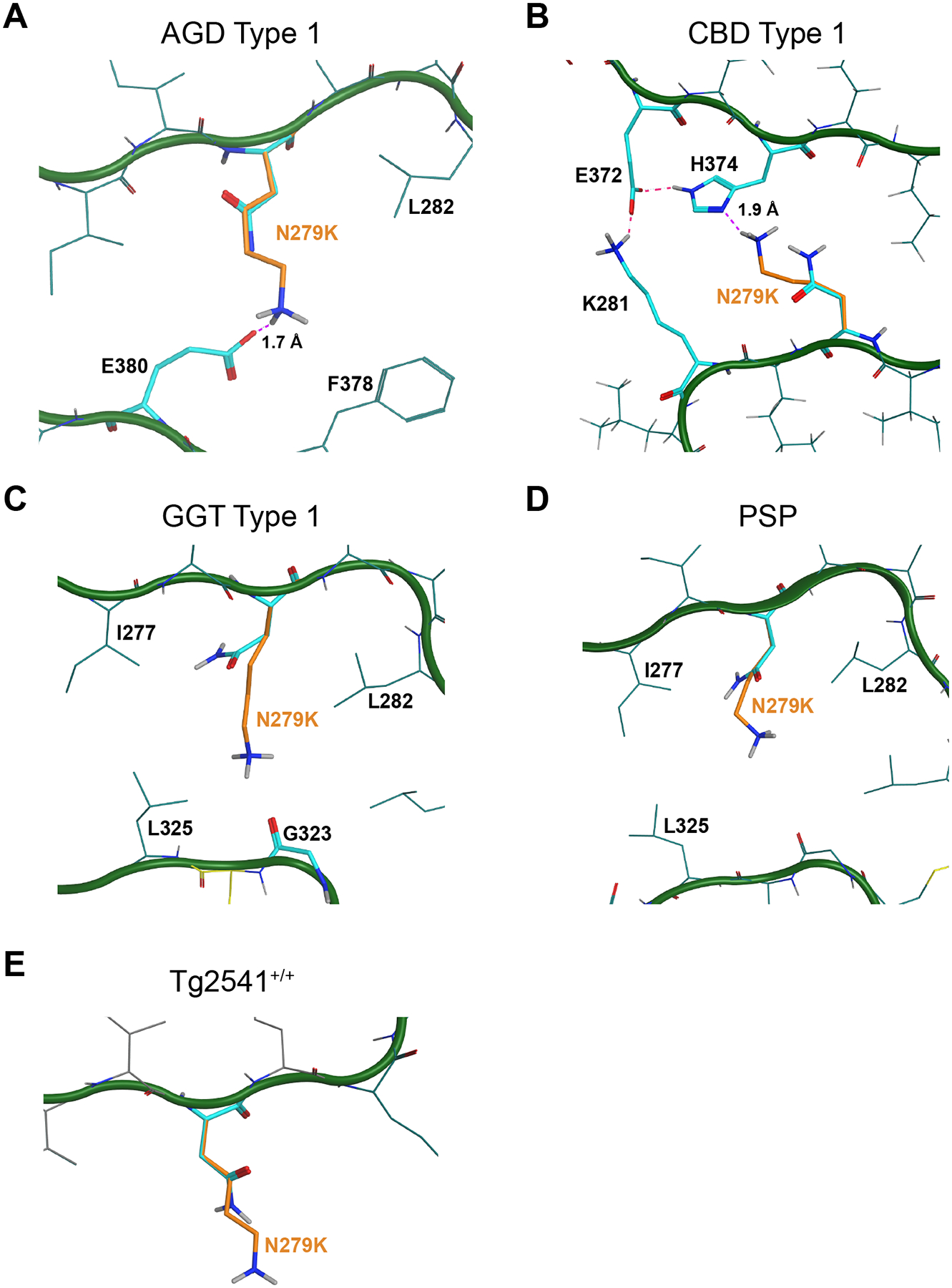
Modeling the N279K mutation on 4R tau cryo-EM structures. MOE software was used to model the effect of the N279K mutation, shown in orange, on tau misfolding into the (A) AGD Type I, (B) CBD Type I, (C) GGT Type I, (D) PSP, and (E) Tg2541^+/+^ tau fibril cryo-EM structures. The carbon backbone is shown in green with the normally occurring residues shown in cyan. (A) In the AGD Type I fibril conformation, the N279K mutation could form a salt bridge with residue E380, based on a predicted distance of 1.7 Å (dotted purple line) between the two residues, however, the hydrophobic pocket surrounding the N279K mutation makes this energetically disfavored. PDB ID: 7P6D. (B) The N279K mutation likely supports tau misfolding into the CBD Type I fibril conformation due to the predicted formation of a salt bridge with H374 over an expected distance of 1.9 Å (dotted purple line). In doing so, an H-bond/salt bridge network forms between E372, K281, H274, and K279 (dotted pink lines), which would further stabilize the conformation. PDB ID: 6TJO. (C & D) In the GGT Type I and PSP fibril structures, the positive charge of the protonated amine in the N279K mutation is not stabilized within the hydrophobic pocket created by I277, L282, and L325. GGT PDB ID: 7P66. PSP PDB ID: 7P65. (E) In the tau fibril structure from the Tg2541^+/+^ mouse model, residue N279 projects outward with no obvious interactions impacted by the N279K mutation. PDB ID: 8Q96.

To understand these data, we modeled the N279K mutation using Molecular Operating Environment (MOE) software (Chemical Consulting Group) in the five cryo-EM structures (Fig. 3). In the CBD fold, there is a moderately sized gap between residues N279, H374, and K281 that can reasonably fit the N279K mutation. Modeling this mutation indicates that the positive charge of the lysine can be neutralized by a salt bridge with H374, and that an H-bond/salt bridge network forms between E372, K281, H374, and N279K, providing strong rationale for the ability of CBD to replicate using tau with the N279K mutation (Fig. 3B). While the AGD fold is similar to the CBD fibril structure in many ways, there is significant divergence at residues 273–283 and 357–383. Although there is open space in the AGD fold to accommodate a lysine at position 279, the hydrophobic gap between L282 and F378 offers no opportunity to neutralize the positive charge. It is possible that N279K could form a salt bridge with residue E380, instead (Fig. 3A), however, due to the hydrophobicity within the pocket, the salt bridge is unlikely to be solvated, creating an increase in the energy requirement to accommodate the N279K mutation. This hypothesis, which is based on static calculations of the overall energetics, is supported by molecular dynamics simulations, which also indicated salt bridge formation is likely disfavored. These measurements are consistent with our *in vitro* data showing AGD tau cannot replicate using the N279K mutation. Notably, the GGT and PSP structures exhibit structural homology around residue N279. Both conformations contain enough empty space to accommodate the N279K mutation, but as was seen with the AGD structure, the residues around the new lysine – L282, I277, and L325 – are extremely hydrophobic. As a result, in both cases, there is no opportunity to mitigate the positive charge of the protonated amine created by the N279K mutation, preventing replication of GGT and PSP tau (Figs. 3C & D). Finally, it is not possible to predict the templating ability of the N279K mutation based on the Tg2541^+/+^ fibril structure. Although the N279 side chain does not have obvious intramolecular interactions (Fig. 3E), one of the two disconnected protein densities in the model has a vector that puts it in close vicinity to residue 279. The inability of Tg2541^+/+^ tau to template in this cell line leads to the hypothesis that the unresolved region of the protein somehow interferes with N279K.

### 2.2. 4R tauopathy samples variably replicate in cells expressing the S285R mutation

We next tested the ability of the 4R tauopathy strains to replicate using the RD of tau containing the S285R mutation (Fig. 2E & F; Supplemental Table 3; representative images shown in Fig. S1). This mutation is also known to increase 4R tau expression through altered RNA splicing in familial FTLD patients (Fujioka et al., 2015; Ogaki et al., 2013). We selected this mutation based on PyMol mutagenesis modeling, which suggested that the longer arginine side chain would cause a severe steric clash with L282 in the CBD conformation. However, when we incubated the Tau4RD*S285R-YFP cells with tau fibrils isolated from the tauopathy patient samples, we found that both the CBD (*P* = 0.0021) and GGT samples (*P* = 0.0051) significantly infected the cell line compared to the negative control samples. The PSP patient samples were also able to infect the cells, though this was not significantly different from control (*P* = 0.08). In contrast, the AGD samples were not capable of replicating using S285R tau (*P* > 0.9999). Data were analyzed using a one-way ANOVA (*F* = 8.011, *P* = 0.0008, DFn = 4, and DFd = 17). Similar to our findings with the N279K mutation, the S285R mutation inhibited replication of the Tg2541^+/+^ mouse sample, though the significant increase compared to the C11 negative control sample (*P* = 0.0318) was not biologically meaningful (Fig. 2F). Normality tests for both datasets are shown in Fig. S2.

**Fig. 4.**
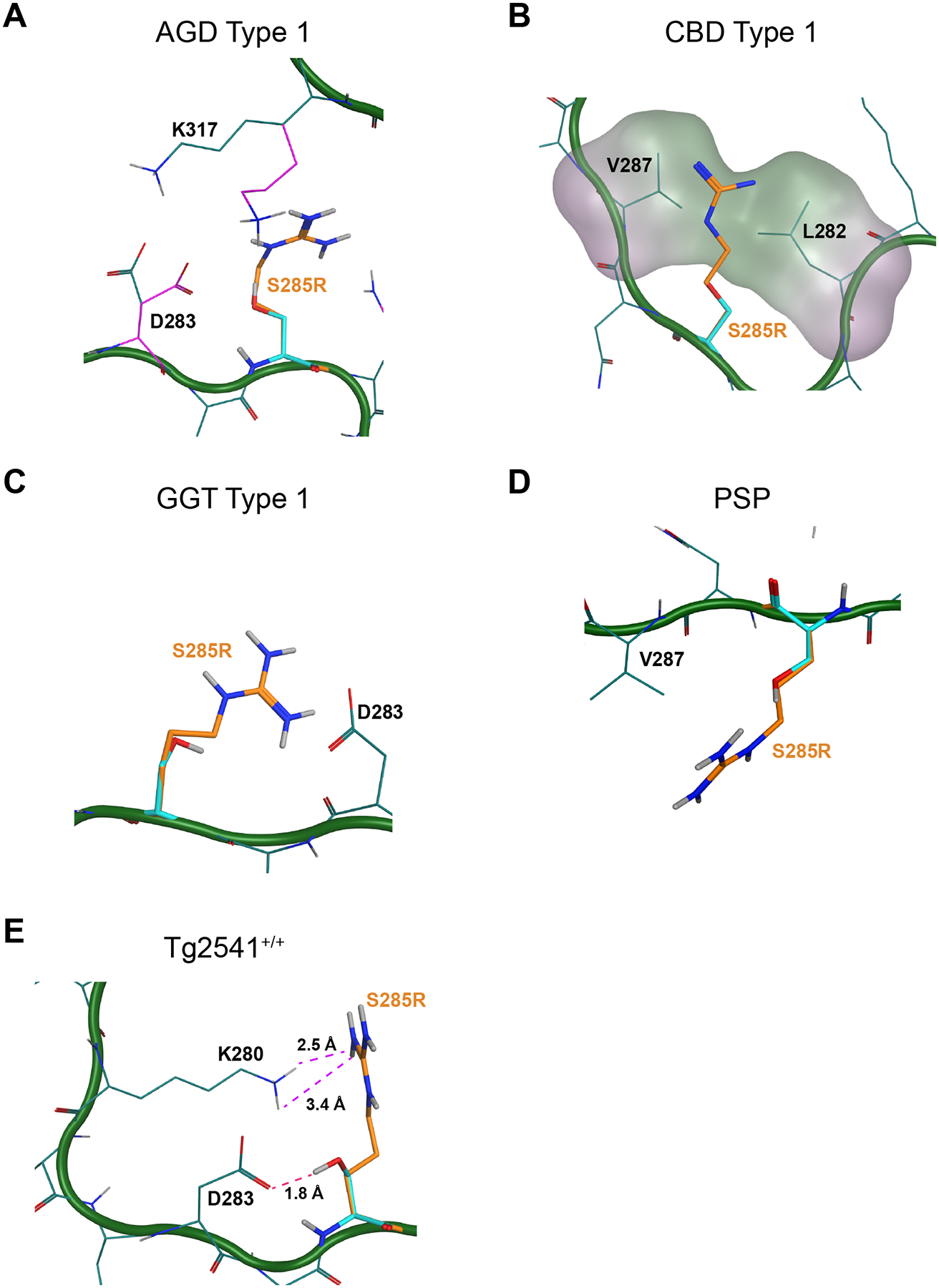
Modeling the S285R mutation on 4R tau cryo-EM structures. MOE software was used to model the effect of the S285R mutation, shown in orange, on tau misfolding into the (A) AGD Type I, (B) CBD Type I, (C) GGT Type I, (D) PSP, and (E) Tg2541^+/+^ tau fibril cryo-EM structures. The carbon backbone is shown in green with the normally occurring residues shown in cyan. (A) In the AGD Type I fibril conformation, accommodating the S285R mutation forces a change in the positioning of D283 and K317 to accommodate the longer side chain. The original position of the two residues is shown in purple alongside the change in positioning in teal. This results in an unmitigated positive charge within the pocket, which would be energetically unfavorable. PDB ID: 7P6D. (B) MOE analysis cannot be used to explain how CBD tau is able to replicate using the S285R mutation. In the absence of deformed templating, it is unclear how the mutation can avoid the predicted clash caused by overlapping van der Waals radii of L282 with V287. S285 sits in a high energy conformation between these residues, suggesting a backbone distortion is required to facilitate replication. PDB ID: 6TJO. (C & D) Residue S285 projects outward into the surrounding solvent in the GGT and PSP structures. GGT PDB ID: 7P66. PSP PDB ID: 7P65. (E) The S285R mutation disrupts a hydrogen bond (pink dotted line) between S285 and D283 (1.8 Å) in the Tg2541^+/+^ fold. Instead, the mutation would place the positive charges on S285R and K280 within proximity of one another (2.5 Å and 3.4 Å interactions shown *via* purple dotted line). PDB ID: 8Q96.

Using MOE to analyze the CBD conformation, we cannot explain how the S285R mutation is able to avoid the predicted clash, apart from the potential occurrence of deformed templating, as has been seen when PrP mutations require a slight change in the PrP^Sc^ packing to accommodate a change in residue size or charge (Makarava and Baskakov, 2012; Makarava et al., 2012). However, we note that upon close analysis, the van der Waals radii of L282 overlap with both K281 and V287, with S285 sitting in a high energy conformation. The Pauli repulsion between these residues indicates that they adopt a slightly different conformation than described in the published model (Fig. 4B). In AGD, we predict that introducing the S285R mutation will force D283 and K317 to adopt new conformations to prevent clashing (original conformation shown in purple and altered conformation shown in teal in Fig. 4A). While this would create space to accommodate the longer R group, it would leave a positive charge within the cavity that cannot be countered by a negative charge, making the conformation energetically unfavorable. Consistent with our *in vitro* data, residue S285 projects outward into the surrounding solvent for both the GGT and PSP structures, enabling tau fibril replication to occur for both strains using mutant monomer (Figs. 4C & D). Intriguingly, in the Tg2541^+/+^ fibril structure, residue S285 also projects outward. However, in this case, the S285R mutation disrupts a hydrogen bond between S285 and D283 and instead positions the positively charged guanidine near the positively charged amine of K280, possibly interfering with templation kinetics (Fig. 4E).

### 2.3. The G303V mutation facilitates 4R tau fibril strain replication

**Fig. 5.**
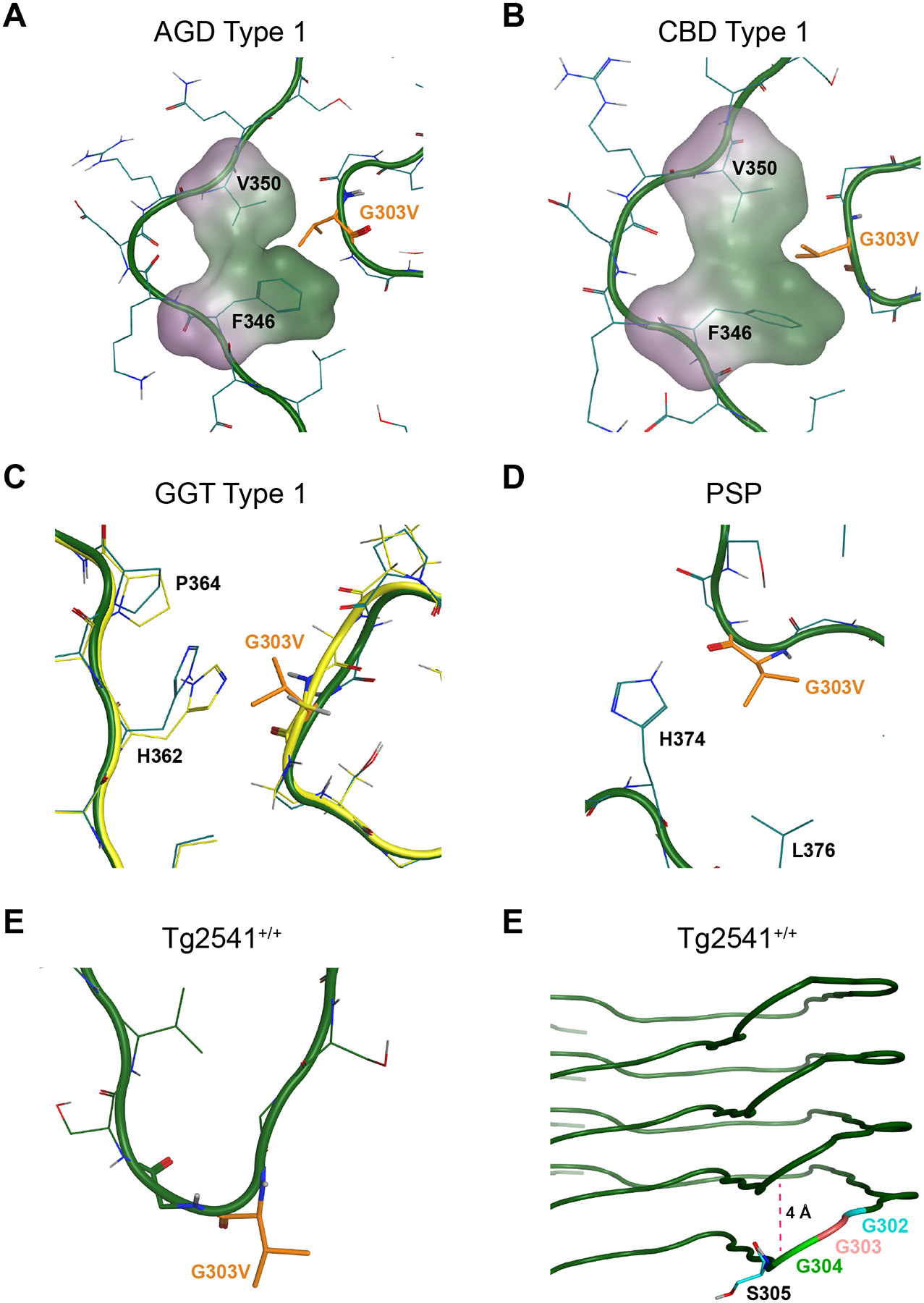
Modeling the G303V mutation on 4R tau cryo-EM structures. MOE software was used to model the effect of the G303V mutation, shown in orange, on tau misfolding into the (A) AGD Type I, (B) CBD Type I, (C) GGT Type I, (D) PSP, and (E) Tg2541^+/+^ tau fibril cryo-EM structures. The carbon backbone is shown in green with the normally occurring residues shown in cyan. (A-D) After minimizing side chains and maintaining backbone rigidity, MOE suggests deformed templating *via* slightly shifting the carbon backbone can prevent steric clashing. (A & B) Space filling models in the AGD and CBD conformations, respectively, demonstrate how G303V can avoid steric clashing at the peak of the turn. AGD PDB ID: 7P6D. CBD PDB ID: 6TJO. (C) Deformed templating to accommodate the G303V mutation in the GGT fibril structure is shown with the initial carbon backbone highlighted in yellow and the altered placement in green. PDB ID: 7P66. (D) The G303V mutation exerts no steric hinderance on PSP tau. PDB ID: 7P65. (E) Residue G303 is located on an outer fold of the Tg2541^+/+^ fibril structure. (F) Mutating the residue to G303V disrupts the planarity of the C_α_ backbone, displacing S305 by nearly 4 Å from the plane of C_α_ carbons between H299 and G302 (dotted pink line). PDB ID: 8Q96.

The G303V mutation, which was identified in early-onset PSP syndrome patients (Choumert et al., 2011; Ros et al., 2005), was selected based on the location of G303 in the known tau fibril structures. Due to glycine having a single hydrogen molecule as its R group, it can tolerate more strain on both the phi and psi angles around C_α_ due to reduced steric hinderance, as is seen in the larger space glycines can occupy on a Ramachandran plot (Ramachandran et al., 1963). Given that G303 is commonly located at an inner curve within the protein fibril fold across several cryo-EM structures, we hypothesized that the G303V mutation would increase steric hinderance around C_α_, preventing some tau strains from replicating *in vitro*. However, we unexpectedly found that all the 4R tauopathy strains tested here were able to replicate in the Tau4RD*G303V-YFP cells, though the PSP infection was not significantly different from control (*P* = 0.0563; Fig. 2G; Supplemental Table 3; representative images shown in Fig. S1). By comparison, infection using the AGD (*P* = 0.0372), CBD (*P* = 0.0117), and GGT samples (*P* = 0.0209) were significantly increased. Data were analyzed using a one-way ANOVA (*F* = 3.646, *P* = 0.0254, DFn = 4, and DFd = 17). While initially counterintuitive to our predictions, using both sidechain minimization and forcing backbone rigidity allows deformed templating to emerge to prevent steric clashing (Fig. 5A–D). This is particularly notable for the GGT structure, where the original structure is shown in yellow and the deformed backbone in green accommodates the G303V mutation to avoid van der Waals overlap (Fig. 5C). Intriguingly, tau fibrils isolated from the Tg2541^+/+^ brain homogenates show an unexpectedly high propensity to replicate in the G303V cell line (Fig. 2H; *P* = 0.0007). This effect is likely because the chain redirection caused by the turn at G303 is located on an outer fold of the Tg2541^+/+^ structure (Fig. 5E) and disrupts the planarity of the C_α_ backbone, displacing S305 by nearly 4 Å from the plane of C_α_ carbons between H299 and G302 (Fig. 5F). Normality tests for both datasets are shown in Fig. S2.

### 2.4. CBD and GGT patient samples replicate in cells expressing the K317H mutation

After evaluating the effects of the familial N279K, S285R, and G303V mutations on 4R tau strain replication, we developed two novel mutations – K317H and D358E – based on PyMol mutagenesis modeling comparing the CBD and PSP cryo-EM structures. We selected the K317H mutation based on our prediction that it points outward to water in the CBD structure and would have no effect on CBD templating (Fig. 1B). Alternatively, PSP has a large positively charged cavity in the center of the fibril containing residues K321, K340, and K317. If a large anionic molecule was to neutralize the charge by binding in the hole, the K317H mutation would change the balance of charge in that region and possibly destabilize binding of the counterion. Empirically testing the 4R tauopathy samples on the Tau4RD*K317H-YFP cell line, we found that only the CBD (*P* < 0.0001) and GGT patient samples (*P* = 0.0046) were able to significantly infect the cells compared to the negative control samples (Fig. 2I; Supplemental Table 3; representative images shown in Fig. S1). The AGD (*P* = 0.7670) and PSP (*P* = 0.9776) samples were unable to replicate in the cells. Data were analyzed using a one-way ANOVA (*F* = 48.09, *P* < 0.0001, DFn = 4, and DFd = 17). In comparison, while the Tg2541^+/+^ brain homogenate showed a statistically increased infection compared to control sample C11 (*P* = 0.0043), this change was not biologically different from control (Fig. 2J). Normality tests for both datasets are shown in Fig. S2.

**Fig. 6.**
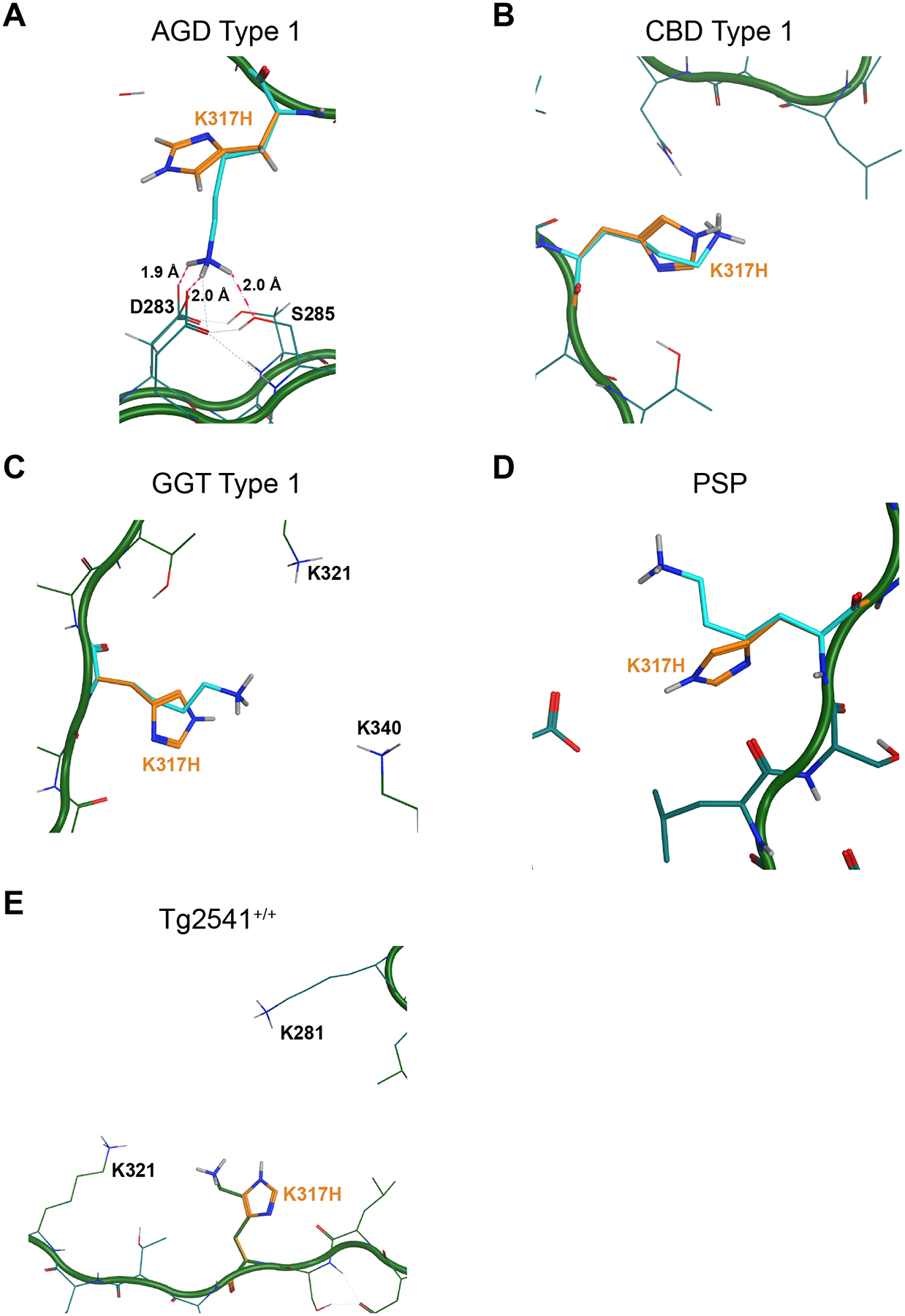
Modeling the K317H mutation on 4R tau cryo-EM structures. MOE software was used to model the effect of the K317H mutation, shown in orange, on tau misfolding into the (A) AGD Type I, (B) CBD Type I, (C) GGT Type I, (D) PSP, and (E) Tg2541^+/+^ tau fibril cryo-EM structures. The carbon backbone is shown in green with the normally occurring residues shown in cyan. (A) In the AGD structure, residue K317 potentially forms a salt bridge with residue D283 or a ternary complex with D283 and S285 (pink dotted lines). The K317H mutation would disrupt this interaction. PDB ID: 7P6D. (B) The K317H mutation projects outward into solvent in the CBD fibril structure, mitigating inhibitory effects on tau misfolding. PDB ID: 6TJO. (C) The GGT structure has a positively charged central cavity defined by lysines at positions 317, 321, and 340. This cavity is filled by an undefined non-protein density (not shown). PDB ID: 7P66. (D) It is unclear from MOE analysis why the K317H mutation has an inhibitory effect on PSP tau propagation. PDB ID: 7P65. (E) Residue K317 also forms a cationic cavity in the Tg2541^+/+^ fold with lysines 281, 317, and 321. The K317H mutation may block fibril extension by disrupting these interactions. PDB ID: 8Q96.

It is difficult to propose a precise mechanism as to why the K317H mutation is unable to propagate tau fibrils isolated from PSP patient samples (Fig. 6D), although as noted above, it is plausible that that an anionic molecule in the central cavity is important for the templating process. Equally fascinating, the GGT structure also has a positively charged central cavity defined by lysines at positions 317, 321, and 340 containing an undefined non-protein density (Fig. 6C). In this case, the hole is smaller and more symmetric. Why the GGT samples can replicate in cells expressing the K317H mutation, but the PSP samples do not is unclear, but it is an interesting area for further research, as it may help identify the nature of the non-protein densities. In the AGD fibril structure, K317 potentially forms a salt bridge with D283 or a ternary complex with D283 and S285 (Fig. 6A). The mutation would likely disrupt this interaction, preventing cell infection. Finally, in the Tg2541^+/+^ fibril structure, K317 forms another cationic cavity, this time defined by lysines as positions 281, 317, and 321. Analogous to the PSP structure, the K317H mutation likely blocks fibril extension by disrupting these interactions.

### 2.5. 4R tau strains variably replicate in cells expressing the D358E mutation

Finally, we tested the effect of the D358E mutation on 4R tau strain replication (Fig. 2K; Supplemental Table 3; representative images shown in Fig. S1). We found that the AGD, CBD (*P* = 0.0136), GGT (*P* = 0.0007), and PSP patient samples (*P* = 0.0044) were able to infect the Tau4RD*D358E-YFP cells, though the AGD sample infection was not statistically different from the negative controls (*P* = 0.8057). Data were analyzed using a one-way ANOVA (*F* = 8.103, *P* = 0.0008, DFn = 4, and DFd = 17). The Tg2541^+/+^ tau prions were unable to replicate using the D358E mutant monomer (Fig. 2L; *P* = 0.0649), which we cannot investigate structurally because residue D358 is not identified in the Tg2541^+/+^ cryo-EM structure (Fig. 1). Normality tests for both datasets are shown in Fig. S2.

**Fig. 7.**
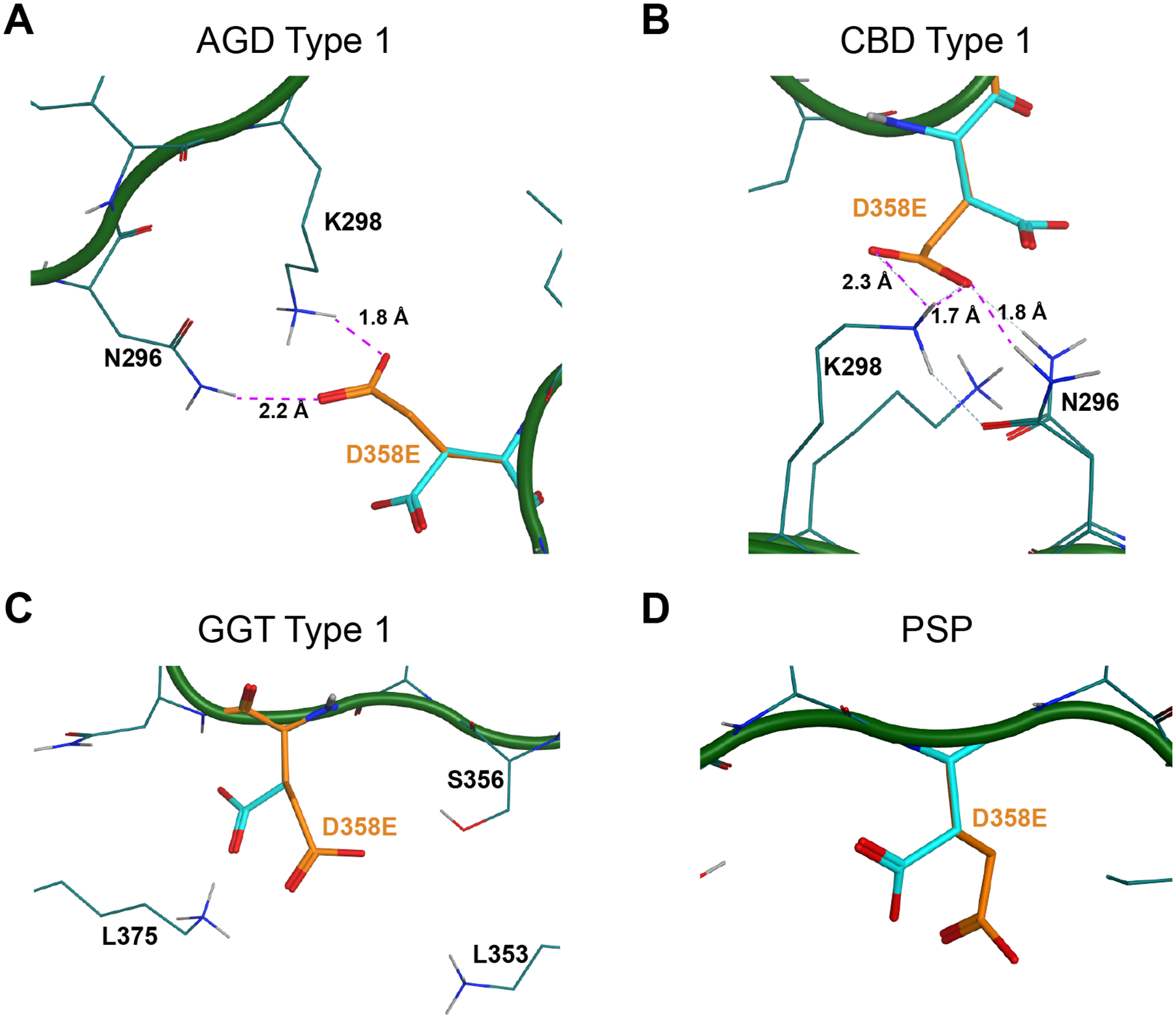
Modeling the D358E mutation on 4R tau cryo-EM structures. MOE software was used to model the effect of the D358E mutation, shown in orange, on tau misfolding into the (A) AGD Type I, (B) CBD Type I, (C) GGT Type I, (D) PSP, and (E) Tg2541^+/+^ tau fibril cryo-EM structures. The carbon backbone is shown in green with the normally occurring residues shown in cyan. (A & B) The D358E mutation in the AGD and CBD folds enables formation of a ternary complex with residues N296 and K298 (dotted purple lines). AGD PDB ID: 7P6D. CBD PDB ID: 6TJO. (C & D) Residue D358 is solvent exposed in the GGT and PSP folds. GGT PDB ID: 7P66. PSP PDB ID: 7P65.

Predicting the effect of D358E on the CBD conformation was also challenging because the β-hydrogens of D358 have a severe van der Waals clash (1.5 Å) with the ammonium hydrogens of K298, and the two residues, unexpectedly, are not predicted to form a salt bridge. Modeling the effect of the D358E mutation in CBD by minimizing each side chain rotamer using MOE for D358E, K298, and N296 reveals a stable ternary complex between the carboxylate of D358E, the amine of K298, and the NH_2_ of N296 (purple dotted line in Fig. 7B). Retrospectively, we propose this as a plausible explanation for the infectivity of CBD after the D358E mutation. Analogous to CBD, we propose that in AGD, the D358E mutation will make a similar ternary complex between D358E, K298, and N296 (dotted purple line in Fig. 7A). In PSP, D358 points out to water where the mutation to glutamate should not affect templating (Fig. 7D), and in GGT, D358 is also solvent exposed (Fig. 7C). The aspartate makes a salt bridge with K375, but modeling with MOE predicts that the longer D358E should readily form a similar salt bridge.

### 2.6. Tau4RD(244–380)-YFP cells differentiate between 4R tau strains

**Fig. 8.**
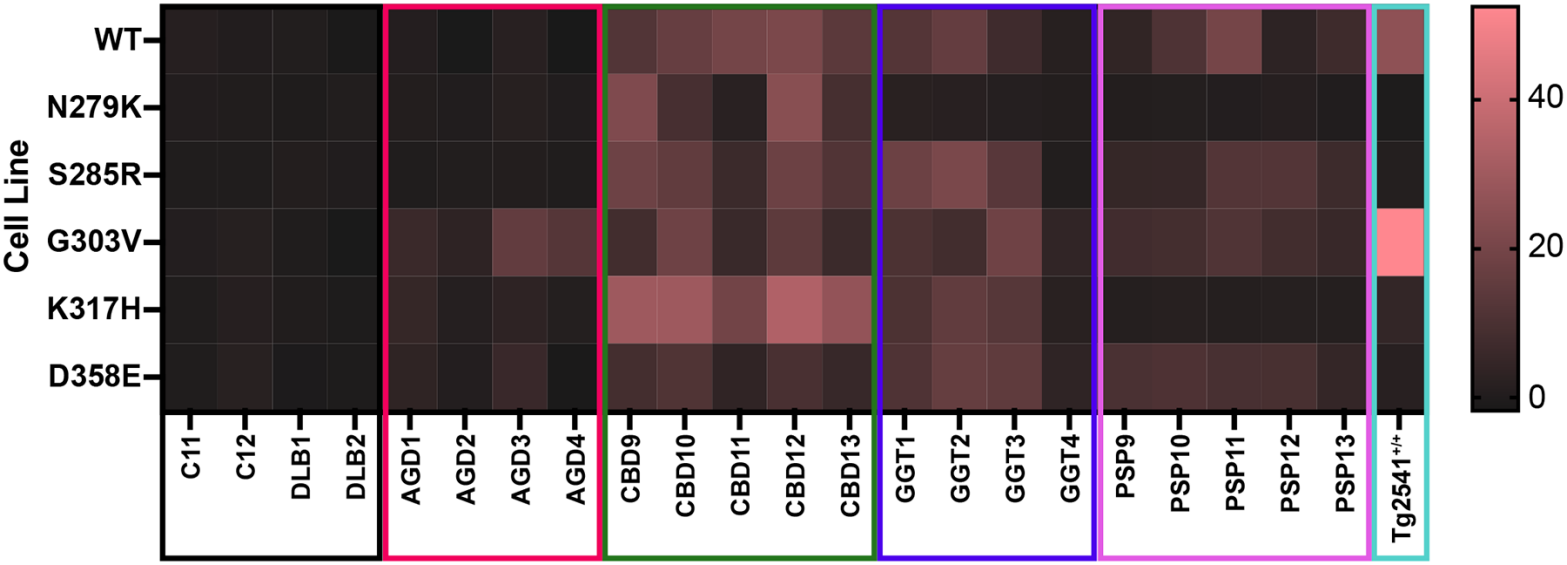
Stratification of 4R tau prion strains by cell infectivity. Heat map representation of data reported in Fig. 2 was generated by normalizing the cell infection value for each patient sample to the average of all 4 negative control samples (C11, C12, DLB1, and DLB2) for each cell line tested. Heat map ranges from black (no infection) to salmon (maximum infection, seen with the Tg2541^+/+^ sample on the G303V cell line). Each patient cohort is characterized by a distinct infectivity profile in the Tau4RD(244–380)-YPF cell lines, with the exception of patient sample GGT4, which most closely aligns with the AGD infectivity profile.

Our experimental goal was to create a panel of cell lines with strain-selective mutations that can be used to distinguish the biological activity of each tau conformation. To determine how well our cell lines can be used to achieve this objective, we generated a heat map showing our infection data for each patient sample tested on each cell line (Fig. 8; raw data reported in Supplemental Table 3). We normalized the data by cell line to the average of the 4 negative control samples, plotting the relative change from control for each sample tested. This resulted in a clustering of patient sample behavior. Specifically, the AGD samples were differentiated by their limited ability to replicate *in vitro*, with the strongest infection in the G303V cells. The CBD samples showed robust infection in each cell line, with the strongest infection in the K317H cells. GGT samples were universally able to infect the Tau4RD (244–380)-YFP cells, with the exception of the N279K cell line. Notably, while samples GGT1, GGT2, and GGT3 all showed similar biological activities, patient sample GGT4 was more consistent with the AGD samples. GGT4 was the only GGT Type III case we tested, which may contribute to this finding. Moreover, GGT Type III is the only subtype of GGT for which no cryo-EM structure is available (Shi et al., 2021). The PSP samples were distinguishable due to their inability to replicate in either the N279K or K317H cells. Finally, the Tg2541^+/+^ pooled sample showed a unique strain biology, with infection strongest in the G303V cells and limited to no infection in the other lines apart from the WT cells. This difference in biological properties from human patient samples is consistent with the distinct structural arrangement of 0N4R tau fibrils isolated from this mouse model (Fig. 1).

## 3. Discussion

Starting with the determination of the tau fibril structure from AD patient samples in 2017 (Fitzpatrick et al., 2017), advancements in cryo-EM have enabled the structural categorization of tau strains by fibril arrangement (Shi et al., 2021), greatly extending beyond our previous studies investigating the specific isoform involvement in each strain (Woerman et al., 2016). While cryo-EM studies have generated an incredible dataset that is highly valuable for better understanding the protein chemistry of tau fibril replication, these data are limited in their ability to definitively assess the biological consequences of each strain. In the studies reported here, we generated Tau4RD(244–380)-YFP cell lines harboring point mutations designed to interfere with tau misfolding into specific conformations. In doing so, we built a cell assay panel that can be used more broadly to identify which tau strain is present in a human patient sample, laying the groundwork for the development of an ante-mortem diagnostic. Moreover, our findings show that tau strain biology in human patient samples differs substantially from the strain biology in the Tg2541^+/+^ mouse model, which is widely used to support basic and pre-clinical research on tauopathies. These results provide compelling evidence that differences in strain biology between rodent models and human patients likely contribute to the challenge of successfully translating anti-tau therapeutics into the clinic.

The last decade has seen significant progress toward the development of ante-mortem biomarkers and diagnostics for tauopathy patients. These efforts include the identification of tau fragments in cerebrospinal fluid (CSF) or plasma (Gonzalez-Ortiz et al., 2023; Horie et al., 2022; Lantero-Rodriguez et al., 2024a; Saijo et al., 2020), changes in CSF or plasma neurofilament light (NfL) levels (Mattsson et al., 2019; Rojas et al., 2018; Yakoub et al., 2023), the presence of various phosphorylated tau species in CSF and plasma (Ashton et al., 2021; Lantero-Rodriguez et al., 2024b; Rojas et al., 2018), and binding of positron emission tomography (PET) probes in the brain (Groot et al., 2024; Nakano et al., 2022; Sanabria Bohórquez et al., 2019; Tagai et al., 2021). However, many of these approaches are broad markers of neurodegeneration. For example, changes in NfL levels are indicative of many neurodegenerative disorders caused by a variety of proteinopathies (Halbgebauer et al., 2022; Oosterveld et al., 2020; Singer et al., 2021), not just the tauopathies. When these approaches can discriminate between tauopathies and other neurodegenerative disorders, they are typically unable to differentiate between disease strains. For example, phosphorylated tau (pTau) species in plasma and CSF have been reported in a variety of tauopathies (Giagkou et al., 2024; Rojas et al., 2018; Schoonenboom et al., 2012; Thijssen et al., 2021), making this a difficult biomarker to rely on for a disease-specific diagnosis. As we continue to learn more about the structural and biochemical underpinnings specific to each tau strain, the importance of developing disease-specific ante-mortem diagnostics becomes increasingly evident.

Previous attempts to develop small molecule therapeutics for prion diseases have shown that compound efficacy can be strain-specific (Berry et al., 2013; Berry et al., 2015; Giles et al., 2017). For example, while the compound IND24 can double the lifespan of mice inoculated with the RML prion strain, the same small molecule is completely ineffective against CJD prions (Giles et al., 2017). Applying these findings to other proteins that rely on the prion mechanism of disease raises the possibility that a therapeutic effective against PSP may yield no clinical benefit for a patient with CBD. Notably, this would reduce the statistical power of a clinical trial, reducing the odds of clinical success. The potential for this outcome suggests a greater need for disease- or strain-specific diagnostics to better facilitate successful clinical intervention for tauopathy patients. This is particularly true in rural communities, where FTLD patients are less likely to receive specialty care and early onset dementias are typically diagnosed and treated by primary care physicians (Xu et al., 2022). In support of this objective, we built a panel of tau reporter cell lines that we successfully used to develop a tau strain-specific signature of the 4R tauopathy strains. The heat map in Fig. 8 demonstrates the ability of this novel tool to differentiate CBD patient samples from other tauopathies based on tau fibril replication in cells expressing the N279K mutation. Comparing GGT and PSP patient samples, tau replication in cells expressing the K317H mutation is in alignment with a GGT Type I or Type II diagnosis whereas lack of replication is consistent with a PSP diagnosis. Finally, low replication in all cell lines except for the G303V cell line is indicative of an AGD diagnosis. While these findings represent a fundamental advancement toward diagnostic development, future efforts will focus on identifying more readily available biofluids (*e.g*., plasma or CSF), rather than post-mortem brain samples, to enable the clinical implementation of these findings.

A critical strength of the approach we report here is the ability to discern key biological differences that exist between each 4R tau strain. This is underscored by the comparison of human tau strain biology with the tau fibrils isolated from terminal Tg2541^+/+^ mice. Using our cellular assay, we found that amplification of Tg2541^+/+^ tau fibrils is blocked when the tau substrate available for replication contains the N279K, S285R, K317H, and D358E mutations, whereas human patient samples were variably able to replicate in each of these cell lines. These biological differences are consistent with the highly distinct cryo-EM fibril structure resolved from Tg2541^+/+^ mouse samples (Schweighauser et al., 2023) compared to the human patient structures (Shi et al., 2021; Zhang et al., 2020), as shown in Fig. 1. While not tested here, tau fibrils isolated from the PS19 mouse model (Yoshiyama et al., 2007) also differ significantly from the 4R tauopathy human patient samples (Schweighauser et al., 2023), suggesting that the tau strain in these mice is also likely to exhibit a unique biological profile in our Tau4RD (244–380)-YFP reporter lines. In reflecting on the failure of IND24 to translate into a successful therapeutic for human prion disease, it is hypothesized that the unique biological properties arising from the distinct structural arrangements of the prion protein in RML and CJD fibrils underlies the strain specificity of the small molecule (Giles et al., 2017). Notably, the PS19 and Tg2541^+/+^ mouse models are both widely used to support preclinical research and therapeutic development for tauopathy patients, raising concerns that the unique fibril structures in the mice may fail to accurately predict human disease biology. Indeed, here we show that the distinct fibril arrangement in Tg2541^+/+^ samples results in altered strain biology compared to the human patient samples. As novel mouse models are developed to support tauopathy research, tools like our cellular assay should be incorporated into model characterization to determine how well each model replicates human disease biology.

While our approach allows us to investigate the biological properties stemming from each unique fibrillar arrangement of tau, we are unable to use these tools to determine the consequence of each strain on cellular biology. Addressing this highly important question requires employing multiple model systems to determine the structure-function relationship underlying tau strain biology. For example, a combination of structural, biochemical, and biological systems is needed to determine how tau misfolding into the CBD conformation causes a unique clinical disease compared to tau misfolding into the PSP conformation. In answering this question, the assay reported here can play an important role in identifying patient samples with unexpected or unique strain biology for further investigation. This is demonstrated by our finding that the only GGT Type III sample in our current dataset, GGT4, exhibited a unique infectivity profile compared to other GGT patient samples. Notably, GGT cases are defined by the presence of widespread globular glial inclusions in the brain, but patients present clinically along a spectrum ranging from behavior variant frontotemporal dementia to motor neuron disease with extrapyramidal features (Ahmed et al., 2013). Given the historical challenges in classifying GGT subtypes (Ahmed et al., 2013), which can be exacerbated by the presence of multiple subtype pathologies in a single patient brain (Kon et al., 2019), the cellular assay developed here offers the ability to stratify GGT patient samples by biological activity. Pairing these efforts with neuropathological investigations and structural data can then be used to further refine disease subtypes, ultimately leading to a more rigorous investigation of subtype-specific disease pathogenesis.

An important limitation to the work reported here is our reliance upon the reported cryo-EM structures to hypothesize why each mutation impacts tau misfolding in a strain-specific manner. As powerful as cryo-EM structures are, the process of resolving each fibril conformation relies on particle picking for the most abundant fibril structure to enable 3D reconstruction. As a result, minor strains or conformations may be missed, as has been shown for α-synuclein strains in patients with multiple system atrophy (Yan et al., 2025). It is also possible that inhibitory effects of each mutation act through alterations in post-translational modifications, which are not seen on the cryo-EM structures, and are therefore not captured in our structural analyses. This would also be true of potential misfolding intermediates that have not yet adopted the mature fibril conformation, though less likely to occur in a seeding-based assay as compared to a spontaneous misfolding model. Intriguingly, in comparing our findings to recent studies using an alanine scan to identify key residues involved with strain-specific tau misfolding (Vaquer-Alicea et al., 2025), we find striking differences in the seeding potential of CBD and PSP tau. While Vaquer-Alicea et al. found that the insertion of alanine mutations in repeats 2, 3, and 4 largely reduced tau monomer incorporation in Tau4RD(LM)-YFP cells seeded with CBD tau, none of the mutations tested in these repeats prevented CBD replication *in vitro* in the studies reported here. In contrast, while the alanine scanning approach found that mutations in R3 were largely responsible for impeding seeding with PSP tau, our results showed the strongest inhibitory effects using mutations N279K in R2 and K317H in R3. This difference may be due to differences in the mutated amino acid used; while alanine is a small hydrophobic residue, the lysine and histidine mutations tested here are positively charged with much longer side chains. As a result, the N279K and K317H mutations may introduce both charge effects and steric hinderance that are less likely to occur with an alanine replacement.

In summary, there is a profound need for model systems that can reliably differentiate tau strains, both for the purpose of supporting diagnostic development and for better identifying and investigating tau strain biology. Using the reporter cell lines described here, we established distinct infectivity profiles across the 4R tauopathy strains underlying AGD, CBD, GGT, and PSP. Moreover, we unexpectedly found that this tool successfully identified a GGT Type III patient sample based on a unique biological signature compared to other GGT samples. This is consistent with the structural differences in GGT Type III that have impeded cryo-EM resolution of tau fibrils isolated from GGT Type III patient samples (Shi et al., 2021). Finally, our studies generate biological evidence supporting the previous structural studies (Schweighauser et al., 2023) indicating that the spontaneous tau fibrils that develop in an animal model of tauopathy are inconsistent with the naturally occurring strains in the human patient population. Together, these data demonstrate the power of our mutagenesis-based approach in building toward an enhanced understanding of the structural consequences of each tau fibril arrangement on disease biology.

## 4. Materials and methods

### 4.1. Human tissue samples

Frozen brain tissue samples from control, AGD, CBD, GGT, and PSP were provided by the University of California, San Francisco (UCSF) Neurodegenerative Disease Brain Bank. None of the patient samples provided were from *MAPT* mutation carriers. Frozen brain tissue samples from neuropathologically-confirmed cases of DLB were provided by the Massachusetts Alzheimer’s Disease Research Center. Detailed information about the patient samples used in this study are available in Supplemental Table 1.

### 4.2. Mice

Animals were maintained in an AAALAC-accredited facility in compliance with the 8th edition of the *Guide for the Care and Use of Laboratory Animals*. All procedures used were approved by the University of California San Francisco Institutional Animal Care and Use Committee. All mice were housed in an ABSL-2 facility in an environmentally controlled room with a 12-h light/dark cycle. Animals had free access to a Tekland diet from Envigo (Indianapolis, IN) and tap water. Mice were group housed unless an animal’s health status necessitated individual housing. The B6-Tg(Thy1-MAPT*P301S^+/+^)2541 mice, or Tg2541^+/+^ mice (Allen et al., 2002), were kindly provided by Dr. Michel Goedert. Aging animals were assessed twice each week for the onset of progressive clinical motor signs. Following the onset of disease, mice were euthanized *via* CO_2_, and the brain was dissected and flash frozen. The brain homogenate aliquot used in these studies was generated by pooling brain tissue from >50 symptomatic mice (both male and female).

### 4.3. Reporter cell line development

Mutant (N279K, S285R, and G303V) human tau cDNA sequences containing residues 244–380 based on the longest 2N4R tau isoform fused to enhanced yellow fluorescent protein by an 18 amino acid flexible linker (EFCSRRYRGPGIHRSPTA) were synthesized and cloned into the pcDNA3.1(+) expression vector by GenScript. The 244–380 peptide fragment was selected over our previous 4RD cell lines (residues 244–378) to encompass the full templating sequence reported in the CBD cryo-EM structure (Zhang et al., 2020). Each mutant sequence was subcloned into the pIRESpuro3 vector (Takara) using restriction cloning with *Eco*RV (5′) and *Not*I (3′). Gene sequence and insertion were confirmed by Sanger sequencing prior to subsequent use. The Tau4RD (244–380)-YFP sequence was generated from one of the mutant sequences using PCR primers designed to correct the point mutation. After confirming the sequence was corrected by Sanger sequencing, the K317H and D358E mutant sequences were generated by PCR amplification of the pIRESpuro3 vector containing the WT sequence using primers designed to introduce each mutation. Sanger sequencing was also used to confirm gene sequence prior to use. Generation of monoclonal HEK293T cells (ATCC) expressing each construct was done as previously described (Reis et al., 2024).

### 4.4. Tau prion bioassay

An Omni Tissue Homogenizer with disposable plastic soft tissue tips (Omni International) was used to make a 10% (wt/vol) homogenate in calcium- and magnesium-free 1× Dulbecco’s phosphate buffered saline (DPBS) using frozen brain samples. Multiple brain homogenates from Tg2541^+/+^ mice were pooled to create the sample tested here. Aggregated protein was isolated from the homogenates using sodium phosphotungstic acid (NaPTA; Sigma-Aldrich) as previously reported (Woerman et al., 2015). Briefly, the 10% homogenates were incubated in 2% (vol/vol) sarkosyl and 0.5% (vol/vol) benzonase at 37 °C with constant agitation in an orbital shaker for 2 h. A final concentration of 2% (vol/vol) NaPTA dissolved in double-distilled H_2_O, pH 7.0, was added prior to incubating the samples overnight in the same conditions. Samples were then centrifuged at 16,000 x*g* for 30 min at room temperature and the supernatant was removed. Pellets were then resuspended in 2% (vol/vol) sarkosyl and 2% (vol/vol) NaPTA prior to incubating for at least 1 h at 37 °C with constant agitation in an orbital shaker. After a second centrifugation step using the same conditions, the supernatant was removed and samples were resuspended in DPBS using 10% of the initial starting volume.

The Tau4RD(244–380)-YFP cell assay was performed in a 384-well plate as previously described (Woerman et al., 2016; Woerman et al., 2017). Optimized assay conditions for each cell line are reported in Supplemental Table 2. After 4 d of incubation, the plate was imaged using the BioTek Lionheart FX automated microscope (Agilent), which collected a DAPI and YFP image from 4 areas per well. The images were analyzed using Gen5 software. First, a primary mask on the DAPI image was used to identify and count nuclei in each image. To ensure accurate quantification, touching objects are split, and object size (minimum and maximum) and fluorescence intensity thresholds were used. Second, a primary mask on the YFP image, using similar thresholding approaches as the DAPI image, was used to identify and sum the total fluorescence in each aggregate. These values are optimized for each cell line using a negative and positive control sample. Values for each of the four images per well were summed to generate one value per well. The YFP fluorescence intensity was then divided by the cell count to normalize infection data for each well. Finally, the average and standard deviation were calculated across the six technical replicates for each sample. Data are reported as fluorescence per cell × 10^5^ arbitrary units (A.U.).

### 4.5. In silico modeling of tau mutations

Molecular Operating Environment (MOE; Chemical Computing Group) software was used for most computational operations. For stability purposes, the build_fibril.svl utility was used to add additional tau subunits to each fibril in the published models. Mutations were performed computationally using the Protein Builder function in MOE, and individual side chain conformations were sampled *via* rotamer search and side chain minimization while keeping the backbone constrained. When side chain conformations showed significant inter-residue interactions, the Maestro (v14.3.129, Schrödinger, Inc., NY, NY) Predict Side Chain task was used to identify sets of low energy rotamers that could be minimized in MOE (Amber EHT) and compared to the MOE-generated conformers.

### 4.6. Molecular dynamics simulations

Molecular dynamics simulations were conducted with Desmond using the Schrödinger suite (v14.3.129). Dynamics setup included explicit water, neutralization, 0.15 N NaCl, and a constraint on each of the backbone atoms to prevent deformation or unraveling. Three discrete simulations were performed (3 × 100 ns) using the NPT ensemble, OPLS5 force field, and TIP3P water model (300*K*). Equilibration utilized a 100 ps NVT ensemble simulation with Brownian dynamics at 10 K, a 12 ps NVT ensemble simulation with a Langevin thermostat at 10 K, two constrained 12 ps NPT simulations using a Langevin thermostat and barostat at 1 atm at 10 K then 300 K, and a 24 ps NPT equilibration using a Langevin thermostat and barostat at 300 K and 1 atm. Membrane relaxation used a 1 ps Nose-Hoover chain thermostat and a 2 ps Martyna-Tobias-Klein barostat. Due to the constraints, stabilization occurred quickly, and side chain conformations were well sampled in the 100 ps time frame. Ten frames from each run were selected from periods of high RMSD stability. Each complex was minimized in Maestro, where conformations, orientations, and energies were compared.

### 4.7. Statistical analysis

Data are presented as mean ± standard deviation. Cell assay data were analyzed using GraphPad Prism software (version 10.1.1). Shapiro-Wilk normality tests were first performed on datasets to determine if a parametric or nonparametric analysis was appropriate (Fig. S2). All human patient sample data were normal, and subsequent analyses were performed using a one-way ANOVA with a Dunnett post-hoc test. Cell assay data comparing the pooled Tg2541^+/+^ brain homogenate with a negative control sample (C11) was done using an unpaired *t*-Test for the WT, N279K, S285R, and G303V cell lines. Data from the K317H and D358E cell lines did not have a normal distribution and were therefore analyzed using a Mann-Whitney test. Significance for all analyses was determined as having a *P* value <0.05.

## Supplementary Material

Supplemental Material

Appendix A. Supplementary data

Supplementary data to this article can be found online at https://doi.org/10.1016/j.nbd.2025.107052.

## Data Availability

All relevant data are within the manuscript and its Supporting information files. Analyzed datasets are available from the corresponding author on reasonable request.
