# Supplementary material for: Tauopathy strains differentially replicate in vitro in the presence of mutant tau monomer
Source: Neurobiol Dis. Author manuscript; Available in PMC 2025 Sep 2. (PMC12403215; doi:10.1016/j.nbd.2025.107052)
Supplement: Supplemental Material [file NIHMS2105450-supplement-Supplemental_Material.docx]

**Online Resource**

Supplementary Material:
Tauopathy strains differentially replicate *in vitro* in the presence of mutant tau monomer

*Neurobiology of Disease*

Christine K. Brown^1 #†^, Matthew P. Frost^1#‡^, Sara A. M. Holec^1,2#^, William W. Seeley^3^, Lea T. Grinberg^3^, Steven H. Olson^4^, and Amanda L. Woerman^1,2*^

^1^Department of Biology and Institute for Applied Life Sciences, University of Massachusetts Amherst, Amherst, MA, USA; ^2^Department of Microbiology, Immunology, and Pathology, Prion Research Center, Colorado State University, Fort Collins, CO, USA; ^3^Departments of Neurology and Pathology, University of California, San Francisco, San Francisco, CA, USA; ^4^Conrad Prebys Center for Chemical Genomics, Sanford Burnham Prebys Medical Discovery Institute, San Diego, CA, USA.

^#^Authors contributed equally to the manuscript.

^†^Current affiliation: Department of Biomedical Engineering, University of Massachusetts Amherst, Amherst, MA, USA.

^‡^Current affiliation: Neuroscience Department, UConn Health, Farmington, CT, USA.

*Corresponding author: Amanda L. Woerman, PhD, Department of Department of Microbiology, Immunology, and Pathology and Prion Research Center, Colorado State University, 300 West Lake St, Fort Collins, CO 80526, [amanda.woerman@colostate.edu](mailto:amanda.woerman@colostate.edu).

**SUPPLEMENTAL FIGURES & FIGURE LEGENDS**

**Figure S1. Representative images of Tau4RD(244–380)-YFP cells infected with tauopathy patient samples.** Tau prions were isolated from control, DLB, AGD, CBD, GGT, and PSP human patient samples, as well as a pooled homogenate from aged Tg2541^+/+^ mice via NaPTA precipitation. Pellets from each sample were incubated for 4 d with cells expressing the Tau4RD(244–380)-YFP construct with either the WT sequences of the N279K, S285R, G303V, K317H, or D358E mutations. Representative images from each cell line incubated with either C11, AGD3, CBD11, GGT2, or PSP11 patient samples, or the Tg2541^+/+^ pooled brain homogenate. YFP signal shown in green. Scale bar, 50 μm.

**Figure S2. Normality tests on Tau4RD(244–380)-YFP cell assay data.** The Shapiro-Wilk test for normality was used to determine if raw data reported in Supplemental Table 3 have a parametric or non-parametric distribution. Graphs show that data collected on the (A & B) WT, (C & D) N279K, (E & F) S285R, and (G & H) G303V cell lines are normally distributed for both (A, C, E, & G) human patient samples and (B, D, F, & H) Tg2541^+/+^ data. By comparison, for both the (I & J) K317H and (K & L) D358E cell lines, the (I & K) human patient sample data have a parametric distribution while the (J & L) comparison between Tg2541^+/+^ and control patient C11 data have a non-parametric distribution.

SUPPLEMENTAL TABLES

Table S1. Patient sample information.

| **Patient** | **Disease** | **Age at Death** | **Sex** | **Brain Region** | **Brain Bank** |
| --- | --- | --- | --- | --- | --- |
| C11 | None | 78 | F | Angular gyrus | UCSF NDBB^a^ |
| C12 | None | 72 | M | Angular gyrus | UCSF NDBB |
| DLB1 | DLB | 81 | M | Anterior cingulate | MADRC^b^ |
| DLB2 | DLB | 68 | M | Anterior cingulate | MADRC |
| AGD1 | AGD | 88 | F | Inferior temporal gyrus | UCSF NDBB |
| AGD2 | AGD | 77 | M | Inferior temporal gyrus | UCSF NDBB |
| AGD3 | AGD | 99 | M | Inferior temporal gyrus | UCSF NDBB |
| AGD4 | AGD | 68 | M | Inferior temporal gyrus | UCSF NDBB |
| CBD9 | CBD | 71 | M | Angular gyrus | UCSF NDBB |
| CBD10 | CBD | 69 | F | Angular gyrus | UCSF NDBB |
| CBD11 | CBD | 79 | M | Angular gyrus | UCSF NDBB |
| CBD12 | CBD | 74 | F | Angular gyrus | UCSF NDBB |
| CBD13 | CBD | 77 | F | Angular gyrus | UCSF NDBB |
| GGT1 | GGT | 80 | F | Middle frontal gyrus | UCSF NDBB |
| GGT2 | GGT | 84 | F | Middle frontal gyrus | UCSF NDBB |
| GGT3 | GGT | 85 | M | Middle frontal gyrus | UCSF NDBB |
| GGT4 | GGT Type III | 82 | F | Middle frontal gyrus | UCSF NDBB |
| PSP9 | PSP | 78 | F | Angular gyrus | UCSF NDBB |
| PSP10 | PSP | 78 | M | Angular gyrus | UCSF NDBB |
| PSP11 | PSP | 71 | F | Angular gyrus | UCSF NDBB |
| PSP12 | PSP | 70 | M | Angular gyrus | UCSF NDBB |
| PSP13 | PSP | 88 | M | Angular gyrus | UCSF NDBB |

# *^a^University of California, San Francisco Neurodegenerative Disease Brain Bank ^b^Massachusetts Alzheimer’s Disease Research Center*

# Table S2. Cell assay experimental conditions.

| **Cell Line** | **Cells per well** | **Lipofectamine (%)** | **Sample dilution in DPBS** |
| --- | --- | --- | --- |
| WT | 2500 | 2.5*^a^* | 1:20 |
| N279K | 3000 | 1 | 1:10 |
| S285R | 3000 | 1.5 | 1:10 |
| G303V | 3500 | 1.5 | 1:10 |
| K317H | 3250 | 1 | 1:20 |
| D358E | 3000 | 1.5 | 1:10*^b^* |

*^a^Tg2541^+/+^sample plated using 2% Lipofectamine 2000.*

*^b^Tg2541^+/+^ sample plated using 1:20 dilution.*

**Table S3. Cell infection data in Tau4RD(244–380)-YFP cells.**

| **Patient ID** | **WT** | **N279K** | **S285R** | **G303V** | **K317H** | **D358E** |
| --- | --- | --- | --- | --- | --- | --- |
| C11 | 4.7 ± 2.5 | 1.3 ± 0.4 | 0.6 ± 0.6 | 4.2 ± 1.9 | 2.2 ± 0.7 | 2.2 ± 3.0 |
| C12 | 3.7 ± 2.7 | 1.2 ± 0.3 | 0.6 ± 0.4 | 4.9 ± 2.1 | 3.5 ± 1.3 | 4.2 ± 2.2 |
| DLB1 | 3.8 ± 1.4 | 0.7 ± 0.1 | 1.3 ± 1.1 | 3.5 ± 1.4 | 2.4 ± 1.5 | 1.6 ± 0.7 |
| DLB2 | 2.1 ± 1.6 | 1.5 ± 0.6 | 0.9 ± 0.6 | 2.1 ± 1.5 | 1.8 ± 0.9 | 1.8 ± 1.4 |
| AGD1 | 4.2 ± 2.3 | 1.5 ± 0.5 | 0.7 ± 0.3 | 9.5 ± 2.7 | 7.1 ± 5.4 | 5.7 ± 8.9 |
| AGD2 | 1.8 ± 0.4 | 1.2 ± 0.3 | 1.1 ± 0.8 | 6.6 ± 4.4 | 3.5 ± 1.6 | 3.0 ± 2.7 |
| AGD3 | 5.1 ± 1.4 | 2.5 ± 0.9 | 1.3 ± 1.1 | 19 ± 14 | 5.4 ± 1.0 | 8.0 ± 4.7 |
| AGD4 | 1.8 ± 1.1 | 1.2 ± 0.4 | 0.7 ± 0.7 | 16 ± 6.9 | 3.2 ± 1.5 | 1.1 ± 1.0 |
| CBD9 | 15 ± 4.6 | 23 ± 14 | 19 ± 7.6 | 11 ± 3.3 | 32 ± 11 | 11 ± 2.0 |
| CBD10 | 20 ± 13 | 10 ± 3.4 | 16 ± 7.9 | 22 ± 6.1 | 32 ± 4.1 | 13 ± 5.1 |
| CBD11 | 23 ± 6.7 | 3.0 ± 1.2 | 7.1 ± 1.0 | 9.3 ± 3.6 | 22 ± 3.8 | 5.9 ± 1.9 |
| CBD12 | 25 ± 14 | 1.8 ± 0.7 | 19 ± 7.9 | 18 ± 5.2 | 36 ± 7.6 | 12 ± 3.2 |
| CBD13 | 17 ± 8.8 | 26 ± 8.1 | 12 ± 2.9 | 10 ± 2.3 | 29 ± 23 | 7.6 ± 0.8 |
| GGT1 | 16 ± 7.8 | 2.9 ± 1.1 | 18 ± 8.6 | 14 ± 4.7 | 13 ± 7.4 | 13 ± 2.1 |
| GGT2 | 19 ± 6.6 | 2.7 ± 1.1 | 22 ± 6.5 | 11 ± 2.8 | 18 ± 6.7 | 18 ± 3.4 |
| GGT3 | 11 ± 3.5 | 2.1 ± 1.0 | 14 ± 7.7 | 22 ± 10 | 15 ± 6.2 | 17 ± 8.8 |
| GGT4 | 4.9 ± 2.7 | 1.6 ± 0.6 | 1.1 ± 0.7 | 7.5 ± 5.2 | 4.3 ± 2.1 | 5.7 ± 1.5 |
| PSP9 | 7.3 ± 2.9 | 1.6 ± 0.7 | 5.5 ± 1.7 | 11 ± 4.5 | 3.3 ± 1.1 | 12 ± 3.4 |
| PSP10 | 14 ± 10.6 | 1.8 ± 1.6 | 5.7 ± 2.4 | 12 ± 2.8 | 3.9 ± 1.5 | 13 ± 5.0 |
| PSP11 | 23 ± 12.4 | 1.7 ± 0.7 | 13 ± 4.3 | 15 ± 2.6 | 3.3 ± 0.9 | 12 ± 5.5 |
| PSP12 | 6.5 ± 5.5 | 2.2 ± 2.2 | 13 ± 4.3 | 11 ± 1.8 | 3.7 ± 0.8 | 12 ± 3.7 |
| PSP13 | 10 ± 6.0 | 1.2 ± 0.6 | 13 ± 2.9 | 9.1 ± 3.4 | 3.4 ± 0.9 | 7.0 ± 2.8 |
| Tg2541^+/+^ | 29 ± 13 | 0.5 ± 0.3 | 1.6 ± 0.7 | 56 ± 26 | 6.7 ± 5.2 | 4.1 ± 1.3 |

*Mean fluorescence/cell ± standard deviation (× 10****^5^****arbitrary units [A.U.]).*
